# Two enzymes contribute to citrate production in the mitochondrion of *Toxoplasma gondii*

**DOI:** 10.1016/j.jbc.2024.107565

**Published:** 2024-07-11

**Authors:** Congcong Lyu, Yanan Meng, Xin Zhang, Jichao Yang, Bang Shen

**Affiliations:** 1 State Key Laboratory of Agricultural Microbiology, College of Veterinary Medicine, Huazhong Agricultural University, Wuhan, Hubei Province, PR China; 2 College of Life Sciences, Longyan University, Longyan, Fujian, PR China; 3 Hubei Hongshan Laboratory, Wuhan, Hubei Province, PR China; 4 Shenzhen Institute of Nutrition and Health, Huazhong Agricultural University, Shenzhen, Guangdong Province, PR China; 5 Shenzhen Branch, Guangdong Laboratory for Lingnan Modern Agriculture, Genome Analysis Laboratory of the Ministry of Agriculture, Agricultural Genomics Institute at Shenzhen, Chinese Academy of Agricultural Sciences, Shenzhen, Guangdong Province, PR China

**Keywords:** citrate synthase, TCA cycle, asexual reproduction, methylcitrate synthase, metabolic flexibility

## Abstract

Citrate synthase catalyzes the first and the rate-limiting reaction of the tricarboxylic acid (TCA) cycle, producing citrate from the condensation of oxaloacetate and acetyl-coenzyme A. The parasitic protozoan *Toxoplasma gondii* has full TCA cycle activity, but its physiological roles remain poorly understood. In this study, we identified three proteins with predicted citrate synthase (CS) activities two of which were localized in the mitochondrion, including the 2-methylcitrate synthase (PrpC) that was thought to be involved in the 2-methylcitrate cycle, an alternative pathway for propionyl-CoA detoxification. Further analyses of the two mitochondrial enzymes showed that both had citrate synthase activity, but the catalytic efficiency of CS1 was much higher than that of PrpC. Consistently, the deletion of CS1 resulted in a significantly reduced flux of glucose-derived carbons into TCA cycle intermediates, leading to decreased parasite growth. In contrast, disruption of PrpC had little effect. On the other hand, simultaneous disruption of both CS1 and PrpC resulted in more severe metabolic changes and growth defects than a single deletion of either gene, suggesting that PrpC does contribute to citrate production under physiological conditions. Interestingly, deleting *Δcs1* and *Δprpc* individually or in combination only mildly or negligibly affected the virulence of parasites in mice, suggesting that both enzymes are dispensable *in vivo*. The dispensability of CS1 and PrpC suggests that either the TCA cycle is not essential for the asexual reproduction of tachyzoites or there are other routes of citrate supply in the parasite mitochondrion.

*Toxoplasma gondii* is an Apicomplexan parasite that invades the nucleated cells of almost all warm-blooded animals (1, 2). The global seroprevalence of toxoplasmosis in humans is estimated to be approximately 25.7% (3). *T. gondii* is also globally distributed in animals, making it a classic zoonotic pathogen. In fact, all human infections originate from animals, by ingestion of undercooked meat that is contaminated with this parasite, or water and fresh produce that contains *Toxoplasma* oocysts shed by infected cats. Although *T. gondii* infection is mostly asymptomatic in healthy individuals, it can cause severe complications to pregnant and immunocompromised humans and animals (2, 4). Currently, the treatment options for toxoplasmosis are limited and no vaccines have been licensed for human use.

As one of the most successful pathogens, *T. gondii* employs a wide variety of strategies to establish parasitic lifestyles in diverse hosts and host cells. Among these is the metabolic flexibility that allows the parasites to satisfy their metabolic needs in different environments. The parasite can utilize various carbon sources like glucose, glutamine, lactate, and even amino acids like alanine to fuel its lytic cycle (5, 6, 7, 8, 9, 10, 11) during the acute phase of infection. As such, the inability to use the main carbon source glucose only led to a 30% reduction in parasite growth (7, 12). However, pyruvate seems to lie at the central node of carbon metabolism and its sufficient production is vital for parasite proliferation. Under normal conditions, pyruvate is mainly produced by the glycolytic enzyme pyruvate kinase 1 (PYK1), which converts glucose or glutamine-derived phosphoenolpyruvate to pyruvate. PYK1 disruption led to severe growth defects, which could be partially rescued by lactate and the rescue was strictly dependent on lactate dehydrogenase 1 (LDH1) to convert lactate to pyruvate (13). It is currently not clear why pyruvate is so critical for parasite growth, as there are multiple metabolic destinations for pyruvate. Like in other eukaryotic cells, pyruvate is transported to the mitochondria of *T. gondii* parasites by the mitochondrial pyruvate carrier (MPC) and then converted to acetyl-CoA by the branched-chain ketoacid dehydrogenase (BCKDH) to enter the TCA cycle. In many apicomplexan parasites like *T. gondii* and *Plasmodium* spp., BCKDH is localized in the mitochondrion to accomplish the function of pyruvate dehydrogenase (PDH), which, in sharp contrast to canonical PDHs, is localized in the parasite apicoplast (14). Deletion of MPC or BCKDH resulted in reduced acetyl-CoA synthesis and parasite growth, suggesting important roles of mitochondrial acetyl-CoA (15, 16, 17). Nonetheless, mutants lacking MPC or BCKDH are viable *in vitro* and virulent in mice, suggesting that either there are other ways to provide acetyl-CoA in the mitochondrion, or the TCA cycle itself is not so critical. Consistent with the latter, conditional knockout of succinyl-CoA synthetase (SCS), a key enzyme in the TCA cycle, resulted in only 30% reduction in parasite growth (18). In addition, deleting six of the eight TCA cycle enzymes did not affect the asexual reproduction of *Plasmodium* parasites in red blood cells. On the other hand, high concentrations of sodium fluoroacetate that inhibited aconitase activity prevented tachyzoite growth (19). The discovery of GABA shunt that allows the conversion of glutamine to succinate explained the lack of a strong growth defect in the SCS depletion mutant (8). Moreover, there are likely additional pathways of acetyl-CoA production in the parasite mitochondrion besides BCKDH, such as β-oxidation of fatty acids, although its activity has not been confirmed experimentally. The inconsistency of these results suggests that the precise role of the TCA cycle in *T. gondii* remains unclear.

Citrate synthase (CS) is a ubiquitous enzyme that catalyzes the first step of TCA cycle by converting acetyl-CoA and oxaloacetate into citrate (20). There are two types of CSs (21). Type I CS proteins form homodimers and are commonly found in eukaryotes, archaea, and gram-positive bacteria (22). In contrast, type II CS proteins are hexamers and are present only in Gram-negative bacteria (23, 24). Although Type I and Type II CS proteins share high sequence similarity, only Type I CS enzymes are regulated by NADH (25, 26, 27). In addition, some microorganisms also express PrpC, a type I CS-like protein involved in 2-methylcitric acid cycle (2-MCC) by converting propionate to pyruvate and succinate (28, 29, 30). To estimate the physiological role of TCA cycle in *T. gondii*, we identified three proteins with putative citrate synthase activities. Subcellular localization studies showed that two of these proteins localized to the mitochondrion. Subsequently, their enzymatic activities and physiological functions were assessed by biochemical and genetic approaches and the results demonstrated that they both contributed to citrate production in the parasite mitochondrion.

## Results

### Identification of putative citrate synthases in *T. gondii*

**Figure 1:**
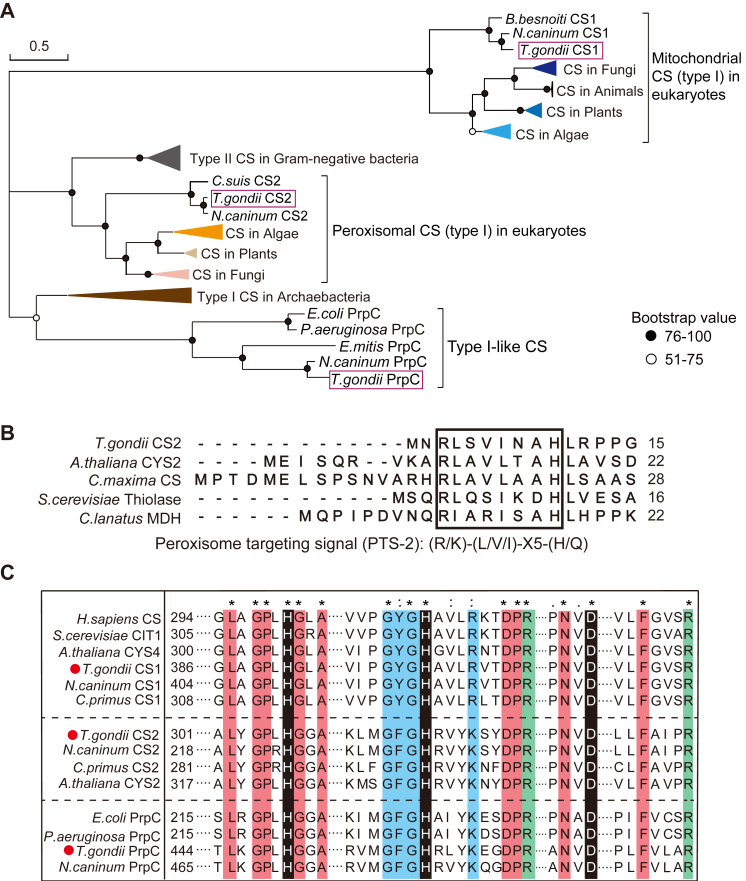
**Phylogenetic relationships and multiple sequence alignment of citrate synthases and related enzymes from diverse organisms.***A*, phylogenetic tree constructed by MEGA 11, using the Maximum Likelihood method based on the LG+G model and the bootstrap value was set as 1000. *B*, the predicted peroxisome targeting signals (PTS2) at the N-termini of selected enzymes, which were shown after aligning indicated proteins by ClustalW. *C*, sequence alignments of CS and related enzymes from selected organisms. The catalytic residues required for CS activity were highlighted in *black*. Key residues for acetyl-CoA/CoA binding were marked in *blue* and those involved in OAA/citrate binding were in *green*.

To look for putative citrate synthases in *T. gondii*, known CS proteins from mammals (*Homo sapiens*), plant (*Arabidopsis thaliana*), and bacteria (*Escherichia coli*) were used as baits to query the *Toxoplasma* genome (https://toxodb.org/) using protein BLAST (pBLAST). Three proteins with sequence similarities to known CS were identified and their gene IDs in the type I strain GT1 are TGGT1_268890 (named CS1 herein, which shares 46.25% amino acid sequence identity with human CS1), TGGT1_203110 (named CS2 herein, which shares 47.43% sequence identity with CYS2 from *A. thaliana*), and TGGT1_263130 (PrpC, which shares 48.13% sequence identity with PrpC from *E. coli*), respectively (Fig. S1). The former two (CS1 and CS2) have also been predicted by a previous study and TGGT1_268890 was predicted to localize to the mitochondrion (18). This previous work also predicted a third putative citrate synthase, but it was not TGGT1_263130. Instead, it was later shown to be the ATP citrate lyase. TGGT1_263130, on the other hand, was thought to be a 2-methylcitrate synthase (PrpC). Existing transcriptomic data in ToxoDB suggest that TGGT1_203110 is probably not expressed or expressed at extremely low abundance in tachyzoites and bradyzoites, but is activated during sexual reproduction stages (Fig. S2). TGGT1_263130 is likely expressed at low levels at most stages. TGGT1_268890, on the other hand, is probably expressed in all stages of the parasite life cycle, although there may be fluctuations in expression levels. To investigate the sequence characteristics and evolutionary relationships of these potential citrate synthases with other proteins, phylogenetic analyses were performed on these proteins, using known CS or CS-like proteins from representative species of mammals, higher plants, algae, archaea, and bacteria (Fig. 1*A*). The results show that the three putative CSs of *T. gondii* clustered into three distinct branches. TGGT1_268890 is grouped with the classic mitochondrial CSs in eukaryotes. TGGT1_203110 is more closely related to CSs found in peroxisomes of plants, fungi, and algae. Consistent with the close phylogenetic relationship between TGGT1_203110 and peroxisomal CSs, TGGT1_203110 also contains a type-2 peroxisomal targeting signal sequence (PTS2) at its N terminus (Fig. 1*B*), suggesting that it may be localized to the parasite peroxisome. On the other hand, TGGT1_263130 is clustered with 2-methylcitrate synthases (also called PrpC) that are involved in the methylcitrate cycle of gram-negative bacteria. Detailed sequence analyses show that all three *Toxoplasma* proteins have the residues that are needed for CS activity, including the catalytic triad, the acetyl-CoA binding motif, and the oxaloacetate/citrate binding motif (Figs. 1*C* and S1). Presence of these sequence signatures suggests that all three proteins are likely to be active CS enzymes. According to these phylogenetic and sequence analyses, we named TGGT1_268890 as CS1, TGGT1_203110 as CS2 and TGGT1_263130 as PrpC.

### *Tg*CS1 and *Tg*PrpC are located in the mitochondrion

**Figure 2:**
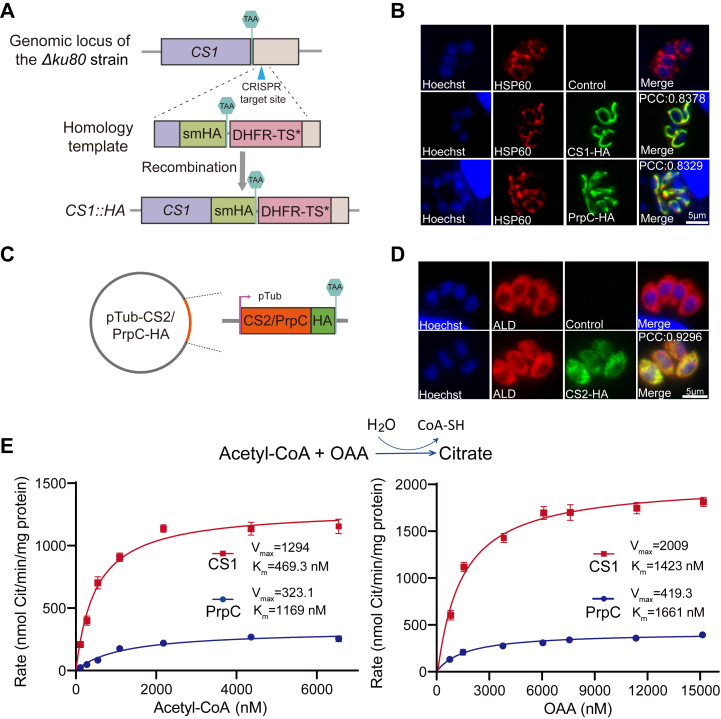
**Two mitochondrion-localized enzymes have citrate synthase activities.***A*, Illustration of the strategy used to insert an smHA tag to the C-terminus of the endogenous *TgCS1* gene, using CRISPR-mediated site-specific integration. *B*, immunofluorescence assays (IFAs) checking the localization of *Tg*CS1-HA and *Tg*PrpC-HA, probed by anti-HA. HSP 60 was included as a mitochondrion-specific marker. *C*, a schematic diagram of the plasmid expressing *Tg*CS2-HA or *Tg*PrpC-HA from the promoter of the Tubulin gene. *D*, IFA on the transgenic parasites expressing *Tg*CS2-HA to estimate the localization of *Tg*CS2. Cytoplasmic *Tg*ALD was stained as a reference. The parental strain without the *Tg*CS2-HA expressing plasmid was used as a negative control. *E*, enzymatic activities of recombinant *Tg*CS1 and *Tg*PrpC purified from *Escherichia coli*. The assays were done with the same batch of recombinant enzyme and each protein was tested three times. Means ± SD were plotted.

To examine the subcellular localization of *Tg*CS1, *Tg*CS2, and *Tg*PrpC in *T. gondii*, the spaghetti monster HA (smHA) tag was fused to the C-termini of target proteins at the corresponding endogenous gene loci (Fig. 2*A*). Subsequently, indirect immunofluorescent assays (IFA) on these transgenic parasites were used to check the subcellular localization of the target proteins, using antibodies against the HA tag. *Tg*CS1 was successfully localized by this approach and it was in the mitochondrion, as evidenced by the overlap of the HA signal with HSP60, a mitochondrion-specific marker for the parasites (Fig. 2*B*). On the other hand, we were not able to detect clear HA signals using the *TgCS2-HA* and *TgPrpC-HA* strains, likely due to the low expression of these two genes at the tachyzoite stage. Alternatively, the coding sequences of *Tg*CS2 and *Tg*PrpC were individually cloned into a vector and expressed in a frame with an HA tag driven by the promoter of the tubulin gene (Fig. 2*C*). After these constructs were introduced into purified tachyzoites, localization of target proteins was determined by IFA. The results showed that *Tg*PrpC co-localized with HSP60, suggesting a mitochondrial localization. On the other hand, *Tg*CS2 was found in the cytoplasm by this approach (Fig. 2*D*), which is in contrast to the PTS2 motif it has. Consistent with this observation, whether *T. gondii* has canonical peroxisomes is still controversial, although proteins containing peroxisomal targeting signals have been identified in its genome. Taken together, of the three proteins with putative CS activities in *T. gondii*, *Tg*PrpC, and *Tg*CS1 are localized in the mitochondrion.

### Both *Tg*CS1 and *Tg*PrpC have citrate synthase activities

To check whether the two mitochondrial proteins have *bona fide* citrate synthase activity, recombinant *Tg*CS1 and *Tg*PrpC proteins were expressed in *E. coli* as His tagged fusion proteins and affinity purified by Ni^2+^ beads (Fig. S3). Subsequently, their CS activities were tested *in vitro* using acetyl-CoA and oxaloacetate as substrates. The results demonstrate that both *Tg*CS1 and *Tg*PrpC could catalyze the condensation of acetyl coenzyme A and oxaloacetate to form citrate. However, the activity of *Tg*CS1 is much higher than that of *Tg*PrpC. Under the same enzyme concentration, the Vmax (therefore the K_cat_) of *Tg*CS1 was 4 ⁓ 4.8 times of that of *Tg*PrpC. In addition, *Tg*CS1 has a higher (about 2.5-fold) affinity for acetyl-CoA than *Tg*PrpC, although their affinity for oxaloacetate is roughly the same (Fig. 2*E*). The higher expression and catalytic activity of *Tg*CS1 over *Tg*PrpC, as well as the closer phylogenetic relationship of *Tg*CS1 with classic mitochondrial CS in eukaryotes all suggest that *Tg*CS1 is the main CS in *Toxoplasma* tachyzoites.

### *Tg*CS1 is important for efficient proliferation of *T. gondii* tachyzoites *in vitro*

**Figure 3:**
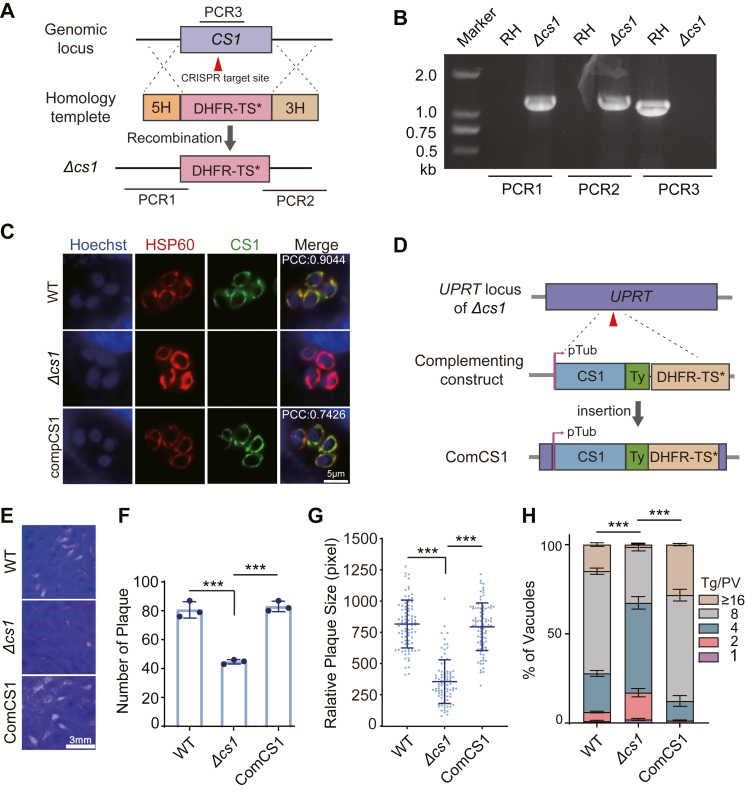
***Tg*CS1 is needed for robust parasite propagation during asexual reproduction.***A*, schematic illustration of *Tg*CS1 deletion by CRISPR/CAS9 mediated homologous gene replacement, which replaced *Tg*CS1 with the selection marker DHFR*-TS*∗ to generate the *Δcs1* strain. *B*, Diagnostic PCRs (PCR1, PCR2, PCR3) on a *Δcs1* clone. *C*, IFA checking the expression of *Tg*CS1 in indicated strains. A home-made mouse anti-CS1 antibody was used to detect *Tg*CS1 and HSP60 was included as a mitochondrial marker. *D*, generation of the *Tg*CS1 complementing strain com*Tg*CS1, by inserting a *Tg*CS1 expressing cassette into the *UPRT* locus of the *Δcs1* mutant. *E–G*, plaques formed by WT (wild type), *Δcs1,* and comCS1 strains. The number and relative size of plaques from *E* are shown in *F* and *G*, respectively. Means ± SD of more than 90 plaques were graphed in G. *∗p* ≤0.05, *∗∗∗p* ≤ 0.001, student’s t-tests. *H*, intracellular replication rates of *Toxoplasma* strains, as indicated by the distribution of parasitophorous vacuoles (PVs) containing different numbers (1, 2, 4, 8, over 16) of tachyzoites after 24 h after of growth. Means ± SEM of three independent experiments, *∗∗∗p* ≤ 0.001, two-way ANOVA with Bonferroni post-tests.

To examine the role of *Tg*CS1 in *T. gondii* tachyzoites, we employed CRISPR/Cas9-mediated gene editing to delete the *Tg*CS1 gene in the type I strain RH (Fig. 3*A*), which was achieved by replacing the *TgCS1* gene with the DHFR-TS∗ selection marker that provided pyrimethamine resistance to the transfectants. Clonal *Δcs1* mutants were identified by diagnostic PCRs (PCR1, PCR2, PCR3). PCR1 and PCR2 examined the insertion of DHFR-TS∗ to the endogenous *TgCS1* locus, whereas PCR3 checked the presence of *TgCS1* gene. The results confirmed the integration of DHFR-TS∗ to replace the endogenous *Tg*CS1 gene in a selected clone (Fig. 3*B*). Deletion of *Tg*CS1 was further confirmed by IFA that probed the expression of the *Tg*CS1 protein (Fig. 3*C*). We also constructed a complementing strain (comCS1) by inserting a CS1-expressing cassette into the *UPRT* locus of the *Δcs1* strain (Fig. 3*D*). To do this, the DHFR-TS∗ selection marker in the *Δcs1* was first removed by introducing the Cre recombinase expressing plasmid p*min-Cre-eGFP* into *Δcs1* tachyzoites and selected the pyrimethamine sensitive clones (*Δcs1*-DHFR*-TS∗*^-^). Then, the com-*TgCS1-TY-DHFR-TS∗* amplicon was inserted into the *UPRT* locus of *Δcs1*-DHFR*-TS∗*^-^ to generate the comCS1 strain. As shown by IFA, the complementing CS1 driven by the promoter of the Tubulin gene (pTub) was successfully expressed in the mitochondrion (Fig. 3*C*). The impact of *Tg*CS1 deletion on tachyzoite growth was first assessed by the plaque assays, which estimate the overall fitness of the parasites. The results showed that the *Δcs1* mutant formed smaller and fewer plaques than the wild-type strain, suggesting critical roles of *Tg*CS1 in tachyzoite growth (Fig. 3, *E–G*). Through a 24 h replication assay, it was found that the *Tg*CS1-deficient mutant exhibited much slower proliferation rates that the parental strain RH (Fig. 3*H*). Additionally, the expression of CS1 in the comCS1 strain was confirmed using a polyclonal antibody against *Tg*CS1 (Fig. 3*C*). As anticipated, the comCS1 strain demonstrated restored efficient reproduction, complementing the effects of *Tg*CS1 deletion (Fig. 3, *E–H*).

### *Tg*PrpC is dispensable for tachyzoite growth *in vitro*

**Figure 4:**
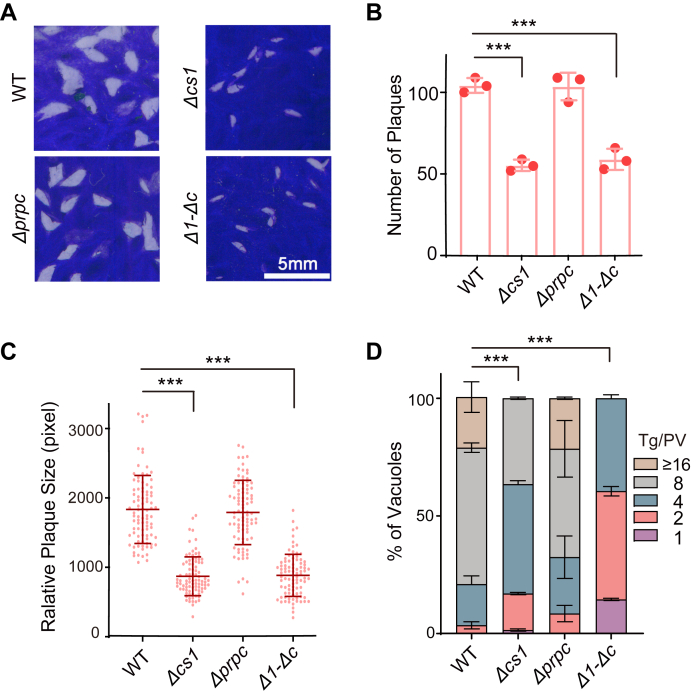
***Tg*PrpC has partial overlapping functions with *Tg*CS1 in promoting tachyzoite proliferation.***A–C*, plaque assays comparing the overall growth of indicated strains. *D*, intracellular replication assays on indicated strains, as done in Figure 3.

Our biochemical analyses indicated that *Tg*PrpC also had CS activity, therefore we sought to determine its role in the parasite. Previous work suggested that *Tg*PrpC was involved in 2-methylcitrate cycle (2-MCC) to detoxify propionate. However, deletion of the 2-methylisocitrate lyase (*Tg*PrpB) that is needed for 2-MCC did not affect parasite propagation both *in vitro* and *in vivo*, suggesting a dispensable role of 2-MCC (28). To explore the physiological functions of *Tg*PrpC, the CRISPR assisted gene deletion strategy was used to knock out *Tg*PrpC in the type I strain RH to generate *Δprpc* (Fig. S4*A*). Diagnostic PCRs confirmed the replacement of the *Tg*PrpC gene by the selection marker DHFR-TS∗ (Fig. S4*B*). Subsequently, we evaluated the growth of the *Δprpc* mutants by plaque and intracellular replication assays. The results showed that the absence of PrpC did not obviously alter the growth or replication of the parasites (Fig. 4, *A–D*). To further check whether there is any functional redundancy between *Tg*PrpC and *Tg*CS1, since both have CS activities, *Tg*PrpC was deleted in the *Δcs1*-*DHFR-TS∗*^-^ strain to create the double knockout strain *Δcs1-Δprpc* (*Δ1-Δc*) (Fig. S5). Phenotypic examinations suggest that the overall growth of the double mutant was similar to that of the *Δcs1* single deletion mutant (Fig. 4, *A–C*), but its intracellular replication rates were further reduced (Fig. 4*D*). These results further support that *Tg*CS1 is the main CS in the mitochondrion of *Toxoplasma* tachyzoites, but *Tg*PrpC probably also has minor roles in generating mitochondrial citrate.

### Both CS1 and PrpC contribute to glucose metabolism and TCA cycle activity

**Figure 5:**
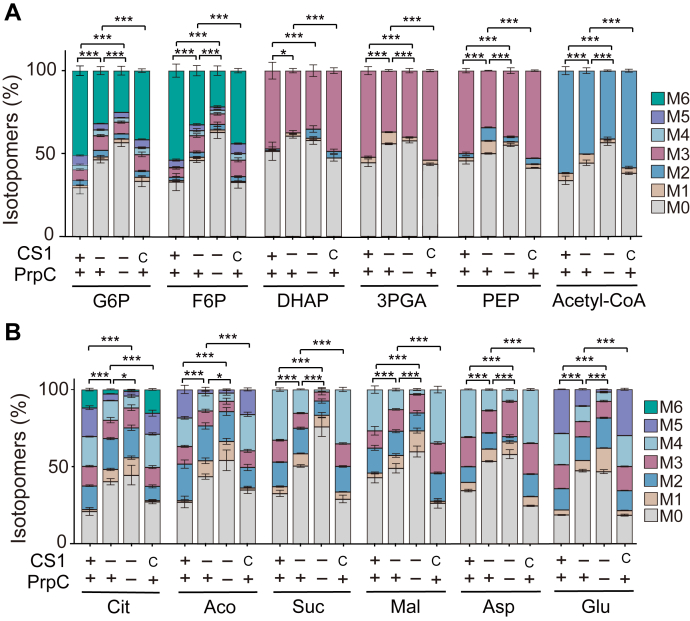
***Tg*CS1 and *Tg*PrpC deletions altered the activities of the central carbon metabolism pathways.***AandB*, Freshly egressed tachyzoites of indicated strains were incubated with 8 mM ^13^C_6_-glucose for 4 h in 37 °C. Then, incorporation of ^13^C into different metabolites (mainly glycolysis and TCA cycle intermediates) were determined by LC-MS. The relative abundance of different isotopomers in each metabolite was plotted. 3PGA, 3-Phosphoglyceric acid; Aco, aconitate; Asp, aspartic acid; Cit, citrate; DHAP, Dihydroxyacetone phosphate; F6P, Fructose 6-phosphate; G6P, glucose 6-phosphate; Glu, glutamic acid; Mal, malate; PEP, phosphoenolpyruvic acid; Suc, succinate. Means ± SD of three independent experiments, *∗p* ≤ 0.05, *∗∗∗p* ≤ 0.001, student’s t-tests. M0-M6 represents the number of carbons in the form of ^13^C in a given metabolite.

Citrate produced by CS1 and PrpC plays a crucial role in the tricarboxylic acid (TCA) cycle. To check if CS1 or PrpC deletions affected the metabolic activities of the parasites, metabolic tracing assays using stable isotope ^13^C labeled glucose were performed. Following established protocols, freshly egressed parasites were incubated with 8 mM ^13^C_6_-glucose for 4 h, and then the metabolites were extracted and quantified by liquid chromatography-mass spectrometry (LC-MS). The incorporation of ^13^C into various metabolites in glycolysis and the TCA cycle was analyzed. The results showed that the incorporation of ^13^C into most glycolysis (including glucose-6-phosphate, fructose 6-phosphate, dihydroxyacetone phosphate, 3-phosphoglyceric acid and phosphoenolpyruvic acid) and TCA intermediates (such as citrate, aconitase, succinate and malate) was significantly reduced in the *Tg*CS1 deletion mutant compared to the parental strain. In addition, the deletion of *PrpC* in *Δcs1* further decreased the flux of glucose-derived ^13^C into glycolysis and TCA cycle (Fig. 5, *A* and *B*). On the other hand, the *Tg*CS1 complementing strain demonstrated normal ^13^C labeling of these metabolites. These results indicate that both CS1 and PrpC contribute to the efficient metabolism of glucose and TCA cycle activity, which is consistent with their roles in citrate production during the TCA cycle. Interestingly, the ^13^C labeling of citrate was reduced instead of completely blocked in the *Δcs1* and the *Δcs1-Δprpc* mutants, suggesting that there might be other pathways to convert glucose-derived carbons into mitochondrial citrate in addition to CS1 and PrpC.

### *Tg*CS1 and *Tg*PrpC deletions only had mild impacts on the virulence of *Toxoplasma* parasites

**Figure 6:**
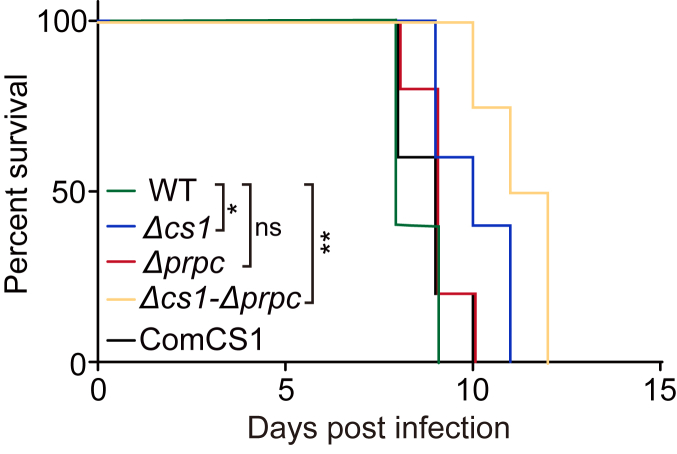
**Virulence of the mutant strains assayed in a mouse infection model.** Purified tachyzoites of the RH, *Δcs1*, *ΔPrpC*, or*Δcs1-ΔPrpC* strains were used to infect ICR mice by intraperitoneal injection. Then the survival of infected animals was monitored daily. Each strain was tested with five mice. *∗p* < 0.05, *∗∗p* < 0.01, Gehan–Breslow–Wilcoxon test.

To assess the impact of *CS1* and *PrpC* deletions on the virulence of *Toxoplasma* parasites, a mouse infection model was used. Mice at the age of 6 to 8 weeks were infected with purified tachyzoites of the wild type, *Δcs1, Δprpc, Δcs1*-*Δprpc* or comCS1 strains at the dose of 100 tachyzoites per mouse. Subsequently, the survival of the infected animals was monitored daily. The results indicate that, although mice infected with any of the tested strains died 8 to 12 days after infection (Fig. 6). The ones infected with *Δcs1* or *Δcs1*-*Δprpc* survived a few days longer. Statistical analyses of the survival curves indicated that the deletion of CS1 slightly, but significantly reduced parasite virulence in mice (*p* value = 0.0177). Similarly, the virulence of the double knockout strain was also modestly reduced (*p* value = 0.0085) (Fig. 6). Therefore, these results suggest that deletion of the major CS proteins in parasite mitochondria did reduce parasite virulence, although the alteration is mild.

## Discussion

*T. gondii* has full TCA cycle activity, but its physiological roles are not fully defined. In this study, we identified three putative CS enzymes that catalyze the first step of the TCA cycle, synthesizing citrate from acetyl-CoA and oxaloacetate. Localization studies demonstrated that two of these enzymes are located in the mitochondrion: *Tg*CS1 and *Tg*PrpC. The third enzyme, *Tg*CS2, which is expressed at an extremely low level, has a peroxisomal targeting signal sequence but is localized in the cytoplasm. Focusing on the two mitochondrial enzymes, our results established that *Tg*CS1 is a canonical mitochondrial CS in eukaryotes and it is responsible for the majority of CS activity in *T. gondii* tachyzoites. *Tg*PrpC was first described as a 2-methylcitrate synthase involved in the 2-methylcitrate cycle. We showed that *Tg*PrpC also had CS activity, although it was much lower than that of *Tg*CS1. Gene deletion and phenotypic analyses suggest that *Tg*CS1 is needed for the rapid proliferation of parasites, but *Tg*PrpC is largely dispensable. Interestingly, the mutants with both *Tg*CS1 and *Tg*PrpC deleted were still viable *in vitro* and virulent *in vivo*, suggesting that either the TCA cycle is not essential for tachyzoite growth, just like the dispensability of TCA in blood-stage *Plasmodium* parasites (31), or, there are other pathways to supply citrate in the mitochondrion. Interestingly, *Toxoplasma* encodes a third CS gene called *CS2* but it is not expressed at the tachyzoite stage, although it has all the sequence signatures for CS activity. In addition, the mRNA levels of *CS*2 did not seem to change significantly after the deletion of both *CS1* and *PrpC* (Fig. S6), suggesting that it may not be able to compensate for the loss of the major mitochondrial CS enzymes, likely due to its cytosolic localization.

While *Tg*CS1 is the major citrate synthase in *T. gondii*, this parasite also has two additional enzymes with putative CS activities. *Tg*PrpC was predicted to catalyze the condensation of oxaloacetate and propionyl-CoA to 2-methylcitrate in the 2-MCC, which is important for the detoxification of propionyl-CoA generated during the catabolism of branched-chain amino acids (29, 30). 2-MCC is commonly found in bacteria and fungi but is absent in mammalian cells, which use the methyl-malonyl-CoA pathway to detoxify propionyl-CoA (32, 33, 34). Sequence analyses did suggest that *Tg*PrpC is closely related to bacteria PrpC proteins, which have sequence similarities to CS proteins and are often clustered as type Ⅰ like CS enzymes. As such, it is not surprising to find that *Tg*PrpC displayed weak CS activity in our enzymatic assays. Interestingly, although deleting *Tg*PrpC in the *Δcs1* mutant did further reduce the flux of glucose-derived carbon into the TCA cycle and glycolysis, it did not arrest parasite growth. These results suggest that while both *Tg*PrpC and *Tg*CS1 are localized in the parasite mitochondrion and are the only known enzymes having CS activities, they have overlapping functions but are not synthetic lethal. This calls into question the essentiality of the TCA cycle in *Toxoplasma* tachyzoites, which is controversial historically. Deletion of a couple of genes related to the TCA cycle, including MPC that imports pyruvate into the mitochondrion and BCKDH that coverts mitochondrial pyruvate into acetyl-coA caused very similar growth defects to tachyzoites as the *Tg*CS1 and *Tg*CS1/*Tg*PrpC deletions (15, 16). Furthermore, depletion of succinyl-CoA synthetase only resulted in 30% growth inhibition, but this could be explained by the GABA shunt that produced succinate from glutamine, another major carbon source for *Toxoplasma* tachyzoites (8, 18). These results suggest that the TCA cycle is important, yet nonessential for tachyzoite proliferation and virulence. On the other hand, the aconitase inhibitor NaFAc (sodium fluoroacetate) severely inhibited tachyzoite growth at high concentrations. However, aconitase has dual localizations in tachyzoites and it was found to localize to both the mitochondrion and apicoplast (35). As such, it is currently not sure whether the inhibition of mitochondrial aconitase that is needed for TCA is responsible for the growth inhibition of parasites by NaFAc. Given the results of MPC, BCKDH, and *Tg*CS1/*Tg*PrpC deletion mutants, it is possible that the fully TCA cycle is indeed not essential at the tachyzoite stage, much like the role of the TCA cycle for blood-stage *Plasmodium falciparum* parasites (31). On the other hand, in *P. falciparum*, inactivation of the lipoate attachment enzyme LipL2, essential for activating the two mitochondrial enzymes BCKDH and α-ketoglutarate dehydrogenase (KDH), entirely blocked parasite growth (36). BCKDH and KDH are synthetic lethal because the acetyl-CoA they generate in the mitochondrion is key for protein acetylation outside of the mitochondrion (36). *Toxoplasma* also has both BCKDH and KDH but their functional relationships are currently unknown.

In addition to the mitochondrial CS1 and PrpC, *T. gondii* encodes a third putative CS named *Tg*CS2 that was localized in the cytoplasm. The expression of *Tg*CS2 was undetectable in tachyzoites and it seems to be oocyst and sporozoite-specific. Although the enzymatic activity of the *Tg*CS2 protein was not determined experimentally, it was expected since it has all the sequence signatures for activity. In addition, phylogenetic analyses indicated that *Tg*CS2 was clustered with peroxisomal CS in other eukaryotes like fungi and plants. Moreover, *Tg*CS2 has a clear PTS2 sequence at the N terminus. As such, peroxisomal localization of *Tg*CS2 is predicted. However, when *Tg*CS2 was fused with an HA tag to examine its subcellular localization, it was found in the parasite cytosol. This is similar to catalase, which also has a peroxisomal targeting signal but is mainly in the cytosol (37, 38). Along with these lines, the presence of peroxisomes in *T. gondii* is still controversial, although many proteins involved in peroxisomal biogenesis and maintenance, as well as classic peroxisomal metabolic pathways like β-oxidation are found in the *Toxoplasma* genome and have PTS motifs, some of these proteins did not show peroxisomal localization (39). It is possible that the presence of peroxisomes may be stage-specific and they exist only in developmental stages like oocysts (40). If this is the case, some of the PTS-bearing proteins may localize to the cytosol or puncta structures in tachyzoites, a stage that lacks *bona fide* peroxisomes. In this regard, the functions of the peroxisome organelle and the proteins it contains deserve further investigation at stages that harbor peroxisomes. In stages that have functional peroxisomes, *Tg*CS2 may localize to this organelle and provide citrate for the parasites through a unique route. Unlike other developmental stages, oocysts and sporozoites are mostly in the environment and can not easily obtain carbon sources and nutrients from the host cells. As such, if peroxisomes are confirmed to be present in oocysts and sporozoites, β-oxidation of fatty acids in the peroxisomes may be an important source of acetyl-coA for the TCA cycle. *Tg*CS2 may convert β-oxidation derived acetyl-coA to citrate to fuel the TCA cycle. These possibilities need to be explored in the future.

## Experimental procedures

### Sequence alignment and phylogenetic analysis

Predicted sequences of *Tg*CS1, *Tg*CS2 and *Tg*PrpC were retrieved from the Toxo DB website (https://toxodb.org/toxo/app/). The orthologs of citrate synthase from verious organisms were identified by BLAST search in NCBI (https://www.ncbi.nlm.nih.gov/) with the amino acid sequence of reported CSs as query sequences. NCBI access numbers are: Mitochondrial CS in parasite: *T. gondii CS1*, EPR64871; *Besnoitia besnoiti CS1*, XP_029219572; *Neospora caninum Liverpool CS1*, XP_003883996; Mitochondrial CS in animals: *H. sapiens CS*, AAQ13428; *Macaca muLatta CS*, EHH20859; *Sus scrofa CS*, XP_020946802; Mitochondrial CS in fungi: *Spizellomyces punctatus* DAOMBR117 *CIT1*, XP_016606675; *Ascobolus immerses RN42 CIT1*, RPA84232; *Saccharomyces cerevisiae S288C CIT1*, NP_014398; Mitochondrial CS in plants: *Secale cereale CS1*, UPO71006; *Oryza sativa CS1*, AAG28777; *A. thaliana CYS4*, NP_001324515; Mitochondrial CS in algae: *Chloropicon primus CS1*, QDZ20183; *Chlorella sorokiniana CS1*, PRW60618; *Trebouxia* sp *CS1,* KAA6419099; Putative peroxisomal CS in parasite: *T. gondii CS2*, EPR61654; *N. caninum Liverpool CS2*, XP_003882425; *Cystoisospora suis CS2*, PHJ25487; Peroxisomal CS in fungi: *Gigaspora margarita CS2*, KAF0555079; *Conidiobolus coronatus CS2*, KXN72308;*Haematococcus lacustris CS2*, GFH18987; Putative peroxisomal CS in algae: *C. sorokiniana CS2*, PRW58871; *C. primus CS2*, QDZ25721;Peroxisomal CS in plants: *A. thaliana CYS2*, OAP06763;*Prunus avium CS2*, XP_021800150; Type I CS in archaebacteria: *Thermotoga maritima MSB8*, AKE28092; *Metallosphaera sedula DSM 5348*, ABP95678; *Saccharolobus solfataricus*, WP_009989299; Type I-like CS in parasite: *T. gondii PrpC*, EPR62453; *N. caninum Liverpool PrpC*, CEL66667; *Eimeria mitis PrpC*, XP_013356372; *E. coli PrpC*, MRF40902; *Pseudomonas aeruginosa PrpC*, WP_128671493; Type II CS in Gram-negative bacteria: *E. coli GltA*, KXL12831; Enterobacteriaceae *GltA*, WP_077128463; *Shigella flexneri GltA*, WP_154839470; *Acetobacter aceti GltA*, AGG68320; *P. aeruginosa GltA*, KXF33600; All sequence alignments were done with Clustal W. Phylogenetic analyses were performed with MEGA 11, using the maximum likelihood method based on the LG+G model with 1000 bootstrap replications.

### HFF cell and parasite culture

The type I strains RH*Δhxgpr*t and RH*Δku80* were used in this study. All transgenic strains were derived from one of these two strains. Human foreskin fibroblasts (ATCC) seeded on plates or flasks were used to culture *T. gondii* parasites. The methods to culture host cells and parasites in DMEM medium supplemented with 2% fetal bovine serum (FBS) (Gibco), 2 mM glutamine, and 1% penicillin–streptomycin were described previously (41).

### Construction of plasmids

All Primers used in this study are listed in Table S1. Locus-specific CRISPR plasmids were constructed by replacing sgUPRT in p*SAG1-Cas9-U6-sgUPRT* (Addgene #54467) with the gRNAs specific-targeting to *Tg*CS1, using site-specific mutagenesis. The plasmids p*CS1::DHFR-TS∗* and p*PrpC::DHFR-TS∗* providing homology donor fragments to replace *Tg*CS1 and *Tg*PrpC genes respectively for the construction of deletion mutants were generated. Briefly, the corresponding 5′- and 3′- homology arms and the DHFR-TS*∗* selection marker were cloned and recombined with the pUC19 vector by multifragment cloning using the ClonExpress MultiS Cloning Kit (Vazyme Biotech). The p*Tub-CS1-TY-DHFR-TS∗* plasmid used to construct the *Tg*CS1 complementation strain was made by replacing *Tg*LDH1 and CAT in p*Com-LDH1* (10) with the coding sequence of *TgCS1* and the selection marker DHFR-TS∗. The p*Tub-CS2-HA-DHFR-TS∗* and p*Tub-PrpC-HA-DHFR-TS∗* plasmids used to check the localization of *Tg*CS2 and *Tg*PrpC were generated by replacing *Tg*MPC1 in p*Tub-MPC1-HA-DHFR*-*TS∗* (16) with *Tg*CS2 or *Tg*PrpC. The pESUMO-CS1(95-559aa) and pESUMO-PrpC(113-619aa) were constructed by replacing *Tg*PFK1 in pE*SUMO-PFK1* (42) with the 95 to 559aa of *Tg*CS1 or the 113 to 619aa of *Tg*PrpC.

### Generation of transgenic parasite lines

To check the localization of *Tg*CS1, a smHA tag was fused to the C-terminus of the endogenous *TgCS1* gene. The cassette containing the smHA epitope and *DHFR-TS∗* selection marker was amplified from the *pSL24m-Linker-smFP-DHFR-TS∗-LoxP-T7* plasmid (43) (homology arms included in primers, Table S1) and co-transfected into RH*Δku80* strain with the CRISPR plasmid p*SAG1-CAS9-sgCS1-loc*, according to previously established protocols (44). Subsequently, the transfectants were selected with 1 μM pyrimethamine (Sigma-Aldrich) for three passages and stable transfectants were then subjected to IFA analyses. To analyze the localization of *Tg*CS2 and *Tg*PrpC, 9.5μg p*Tub:CS2-HA-DHFR-TS∗* or p*Tub:PrpC-HA-DHFR-TS∗* plasmids were mixed with 1 × 10^6^ cells in cytomix buffer and then electroporated into RH *Δku80* strain. Transfected parasites were then used to infect HFF cells and cultured for 16 h at 37 °C and 5% CO_2_ before IFA analyses.

To make the *Tg*CS1 or *Tg*PrpC deletion mutants, homology templates CS1::DHFR-TS*∗* or PrpC::DHFR-TS*∗* amplified from p*CS1::DHFR-TS∗* and p*PrpC::DHFR-TS∗* respectively were transfected into tachyzoites of the RH*Δhxgprt* strain along with gene-specific CRISPR plasmids. Transgenic parasites were selected with 1 μM pyrimethamine and single clones were examined by diagnostic PCRs (PCR1/PCR2/PCR3). PCR1 and PCR2 examined the insertion of the selection marker to the endogenous locus of the target gene, whereas PCRs examined the presence of the target gene. The*Δcs1*-*DHFR-TS∗*^-^ strain was obtained by transfecting the p*min-Cre-eGFP* plasmid into the *Δcs1* strain. Subsequently, single clones were individually examined by pyrimethamine treatment and diagnostic PCRs. Only pyrimethamine-sensitive clones with the right diagnostic PCRs confirming the removal of DHFR-TS∗ were kept for downstream use to construct the double deletion mutant *Δcs1-ΔPrpC* and complementing strain comCS1. The *Δcs1-Δprpc* mutant was generated by replacing *Tg*PrpC in the *Δcs1-DHFR-TS∗*^-^ strain with DHFR-TS∗, using similar methods as the construction of *Δcs1* described above. The complementation strain comCS1 was constructed by transfecting the p*SAG1:Cas9-U6:sgUPRT* plasmid and the p*Tub:CS1-TY-DHFR*-*TS∗* fragment amplified from the p*Tub:CS1-TY-DHFR-TS∗* plasmid into the *Δcs1*-*DHFR*-*TS∗*^-^ strain. Subsequently, the transfected parasites were selected with 1 μM pyrimethamine and single clones were examined by diagnostic PCRs and IFA before use.

### Parasite growth and replication assays *in vitro*

The 24 h replication assay and plaque assay were performed to monitor the growth of parasite. To estimate the intracellular replication rates of parasites, freshly egressed parasites were purified by filtration through 3.0 μm membranes, and then used to infect HFF monolayers seeded on coverslips for 15 min. Uninvaded parasites were washed away with PBS three times and the invaded ones were allowed to grow for 24 h at 37 °C. Subsequently, the samples were fixed and stained with 4% paraformaldehyde. The number of parasites in each parasitophorous vacuole was determined under an Olympus BX53 fluorescence microscope (Olympus Life Science, Japan). To estimate the overall growth of parasites by plaque assays, freshly egressed parasites were purified, counted, and used to infect HFF cells seeded in 6-well plates. Then the cells were cultured at 37 °C for 7 days without movement. Samples were then fixed with 4% paraformaldehyde and stained with crystal violet to visualize the plaques (45). The plates were subsequently scanned with MICROTEK scanner and the size of the plaques was analyzed by Adobe Photoshop 2018.

### Indirect immunofluorescence assay

Indirect immunofluorescence assays (IFA) were performed according to previously described protocols (42). The following primary antibodies were used: mouse anti-HA (Medical & Biological Laboratories Co), mouse anti-TY, rabbit anti-*Tg*HSP60, and rabbit anti-*Tg*ALD. Alexa-488 or Alexa-594-conjugated were used as secondary antibodies (Life Technologies). Hoechst 33342 (Beyotime) was used to stain the nuclei. Stained samples were imaged by an Olympus BX53 fluorescence microscope (Olympus Life Science).

### Metabolite extraction and metabolic labeling of parasite

To detect the metabolic flux through glycolysis and TCA cycle, 3 x 10^7^ fresh egressed tachyzoites were resuspended in DMEM medium containing 8 mM ^13^C_6_-glucose at 37 °C for 4 h. Subsequently, samples were washed with glucose-free DMEM and PBS, and then resuspended in 80% aqueous methanol. Metabolites were extracted and quantified by UHPLC-HRMS (Ultra-High-Performance Liquid Chromatography-High-Resolution Mass Spectrometry). Each strain was tested three times independently, from metabolite extraction to mass spec analyses.

The LC-MS analyses were performed on the Dionex Ultimate 3000 UPLC system coupled to a TSQ Quantiva Ultra triple-quadrupole mass spectrometer (Thermo Fisher), equipped with a heated electrospray ionization (HESI) probe. Extracted metabolites were separated by a synergi Hydro-RP column (2.0 × 100mm, 2.5 μm, phenomenex). A binary solvent system was used, in which mobile phase A consisted of 10 mM tributylamine (pH adjusted with 15 mM acetic acid) in water and mobile phase B of methanol. The analysis used a 25-min gradient from 5% to 90% mobile B. Data were acquired in selected reaction monitoring (SRM) in a positive-negative ion switching mode. The resolution for Q1 and Q3 were both 0.7 FWHM. The source voltage was 3500v for the positive and 2500v for the negative ion mode. The source parameters are as follows: capillary temperature: 350 °C; heater temperature: 300 °C; sheath gas flow rate: 35; auxiliary gas flow rate: 10. Tracefinder 3.2 (Thermo) was applied for metabolite identification and peak integration.

### Protein purification and enzymatic assay

The expression and purification of *Tg*CS1 and *Tg*PrpC were performed according to previously reported protocols (46). The p*ESUMO-CS1* or p*ESUMO-PrpC* plasmids were transformed into Transetta (DE3) cells individually. For the expression of the recombinant proteins, was induced with 0.2 mM IPTG for 8 h at 18 °C with shaking at 180 rpm. Subsequently, the bacterial cells were lysed with a French press and the recombinant proteins were purified on a Ni^2+^ column, following the instructions from the manufacturer. The purified proteins were dialyzed in PBS buffer and the final concentrations were determined with a BCA protein assay kit (Beyotime, China). Then, the enzymatic activities of CS1 and PrpC were measured using a commercial citrate synthase activity assay kit (Solarbio, China). The reactions were performed in 100 μl (containing 0.7 μg enzyme in 3.5 μl, 3.5 μl OAA, and 3.5 μl acetyl-CoA at various concentrations) in 96-well plates, according to the instructions from the manufacturer. Then OD_412_ values of the reaction samples were recorded for 10 min with 20 s intervals in the 96-well plate reader. The enzymatic activities of recombinant proteins were determined by the following equation: $\text{CS}\;\text{activity}\;\left(U/\text{mg}\;\text{prot}\right)=\Delta_{\text{OD}412}÷\left(\varepsilon \times d\right)\times V\;\left(\text{total}\;\text{reaction}\;\text{volume}\right)÷(\text{Cpr}\times V\;\left(\text{tested}\;\text{protein}\;\text{volume}\right))÷T.\;\varepsilon =13.6\times 10^{-3}\text{ml}/\left(\text{nmol}\cdot \text{cm}\right)$, Cpr: concentration of enzyme. d, 96-well plate optical path (0.6 cm) V, volume; T, reaction time.

### Parasite virulence in mice

Six to eight weeks old female ICR mice were used to test the virulence of *T. gondii* strains. Freshly egressed tachyzoites were collected, purified, and counted to infect mice by intraperitoneal injection. Each strain was tested with 5 mice and each was infected with 100 purified tachyzoites of indicated strains through intraperitoneal injection in 200 μl. The mice were then monitored daily for their symptoms and survival. All animal experiments were approved by the ethical committee of Huazhong Agricultural University (permit number HZAUMO-2022-0183).

### Data analysis and statistics

All assays shown were repeated at least three times independently unless stated otherwise. Statistical analyses were performed in GraphPad Prism 8.0.2 (GraphPad Software Inc.) using the Gehan–Breslow–Wilcoxon test, two-way ANOVA with Bonferroni post-tests or Student’s *t* test.

## Data availability

All data generated in this study are provided in the article and its supplementary materials.

## Supporting information

This article contains supporting information.

## Conflict of interest

The authors declare that they have no conflicts of interest with the contents of this article.
