# Supplementary material for: Two enzymes contribute to citrate production in the mitochondrion of Toxoplasma gondii
Source: J Biol Chem. 2024 Jul 11;300(8):107565. doi: 10.1016/j.jbc.2024.107565 (PMC11359734; doi:10.1016/j.jbc.2024.107565)
Supplement: Supplemental Figure S2 [file mmc2.pdf]

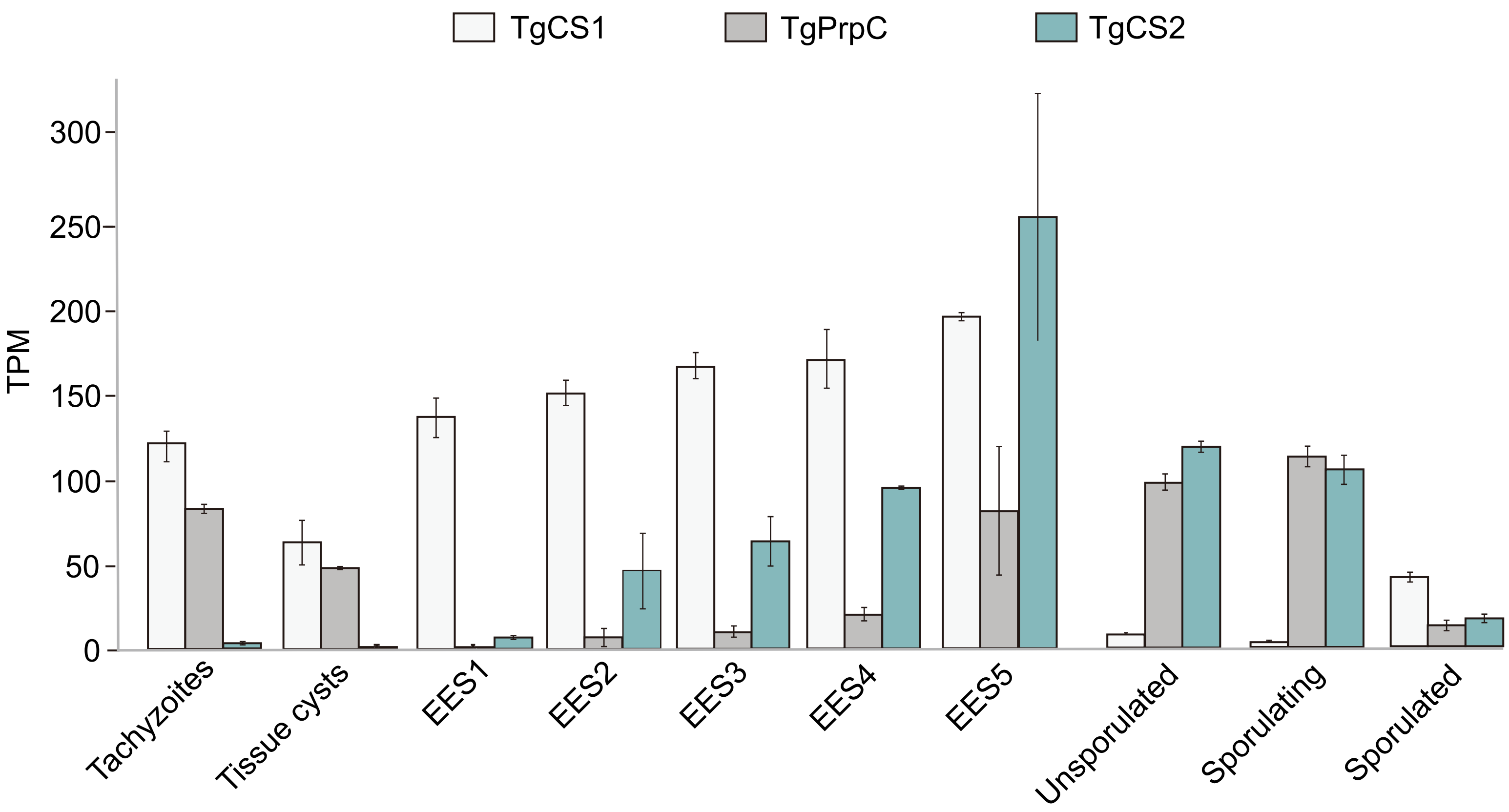

Figure S2. Transcript levels of TgCS1, TgPrpC, and TgCS2 across various life cycle stages of Toxoplasma gondii. TPM: Transcripts Per Million, EES1 = very early enteroepithelial stages in cats; EES2 = early enteroepithelial stages in cats; EES3 = mixed enteroepithelial stages in cats; EES4 = late enteroepithelial stages in cats; EES5 = very late enteroepithelial stages in cats.These data are retrieved from the ToxoDB database.
