# Supplementary material for: Two enzymes contribute to citrate production in the mitochondrion of Toxoplasma gondii
Source: J Biol Chem. 2024 Jul 11;300(8):107565. doi: 10.1016/j.jbc.2024.107565 (PMC11359734; doi:10.1016/j.jbc.2024.107565)
Supplement: Supplemental Figure S3 [file mmc3.pdf]

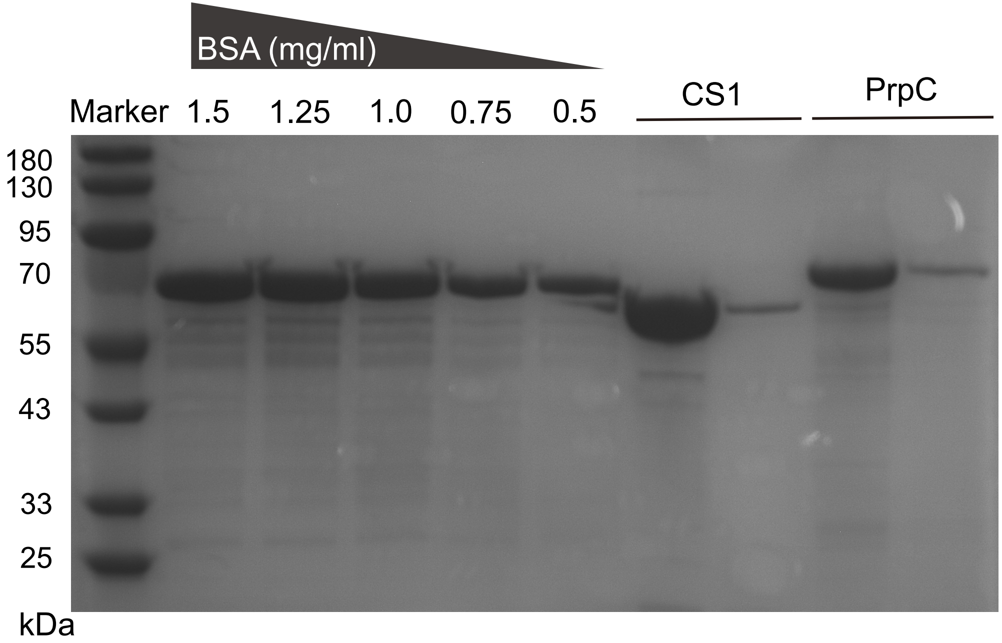

Figure S3. SDS-PAGE analysis of purified recombinant CS1 and PrpC proteins. The purity and concentration of the recombinant proteins were assessed. Bovine serum albumin (BSA) standards at the indicated concentrations were included for estimating the concentrations of CS1 and PrpC.
