# Supplementary material for: Two enzymes contribute to citrate production in the mitochondrion of Toxoplasma gondii
Source: J Biol Chem. 2024 Jul 11;300(8):107565. doi: 10.1016/j.jbc.2024.107565 (PMC11359734; doi:10.1016/j.jbc.2024.107565)
Supplement: Supplemental Figure S4 [file mmc4.pdf]

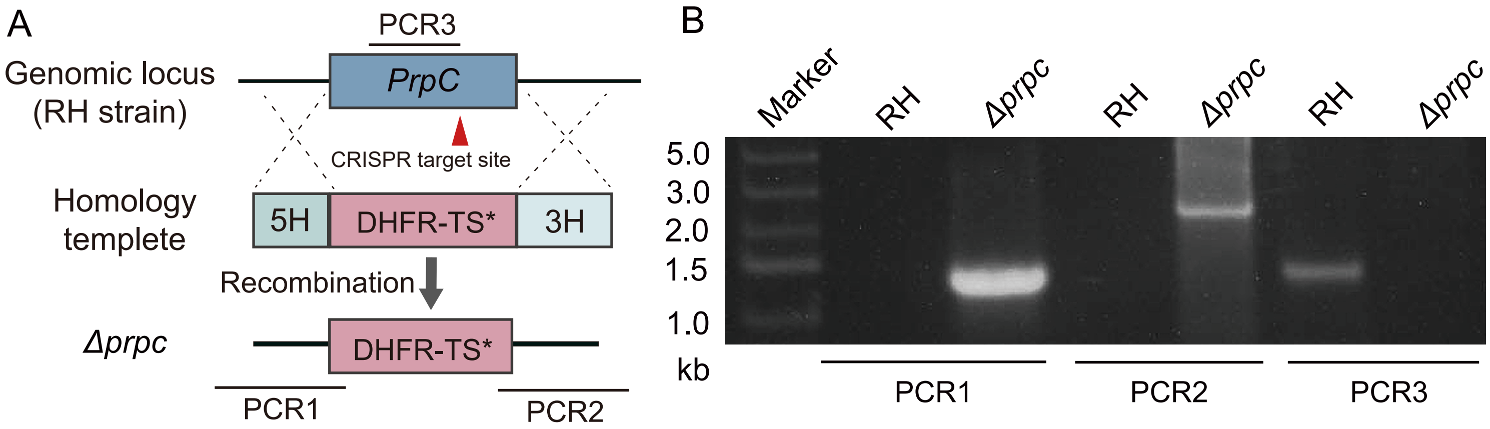

Figure S4. Construction of the *Δprpc* strain. A, Schematic illustration of *Tg*PrpC deletion. *Tg*PrpC was deleted by CRISPR/CAS9 mediated homologous gene replacement, which replaced *Tg*PrpC with the selection marker DHFR-TS* to generate the *Δprpc* strain. B, Diagnostic PCRs (PCR1, PCR2, PCR3) on a *Δprpc* clone.
