# Supplementary material for: Two enzymes contribute to citrate production in the mitochondrion of Toxoplasma gondii
Source: J Biol Chem. 2024 Jul 11;300(8):107565. doi: 10.1016/j.jbc.2024.107565 (PMC11359734; doi:10.1016/j.jbc.2024.107565)
Supplement: Supplemental Figure S5 [file mmc5.pdf]

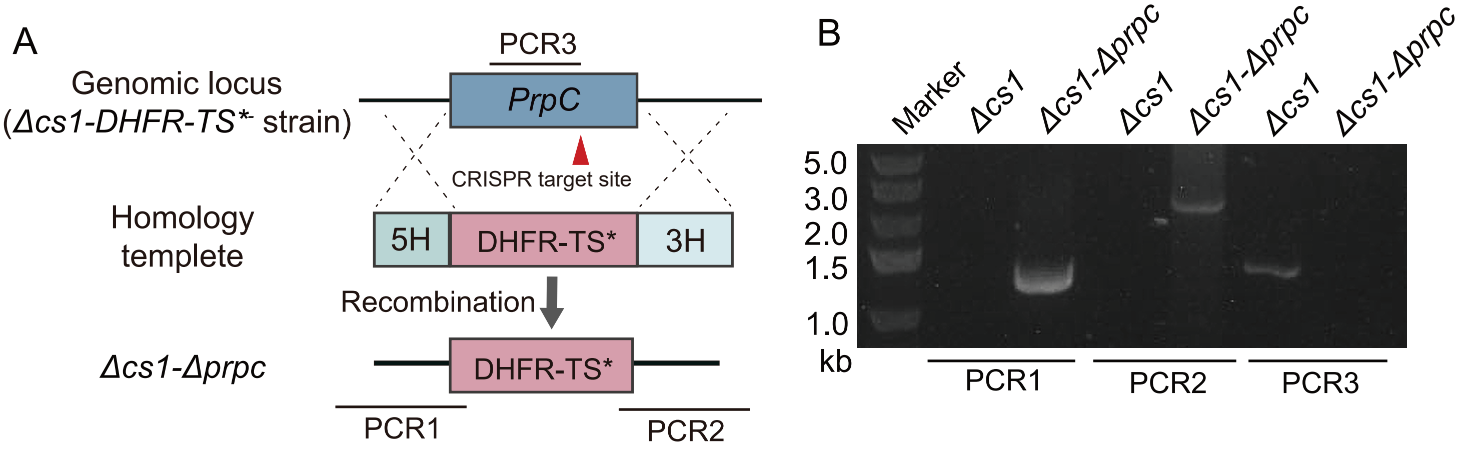

Figure S5. Construction of the *Δcs1-Δprpc* strain. A, Schematic illustration of *TgPrpC* deletion in the *Δcs1-DHFR-TS*-* strain (*Δcs1*). *TgPrpC* was deleted by CRISPR/CAS9 mediated homologous gene replacement, which replaced TgPrpC with the selection marker *DHFR-TS** to generate the *Δ cs1-Δprpc* strain. B, Diagnostic PCRs (PCR1, PCR2, PCR3) on a *Δcs1-Δprpc* clone.
