# Supplementary material for: Two enzymes contribute to citrate production in the mitochondrion of Toxoplasma gondii
Source: J Biol Chem. 2024 Jul 11;300(8):107565. doi: 10.1016/j.jbc.2024.107565 (PMC11359734; doi:10.1016/j.jbc.2024.107565)
Supplement: Supplemental Figure S6 [file mmc6.pdf]

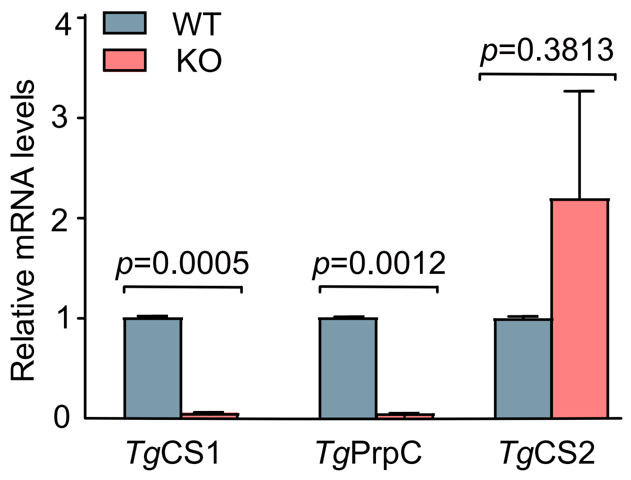

Figure S6. Relative transcription levels of *TgCS1*, *TgCS2* and *TgPrpC* in wild-type (WT) and *Δcs1-Δprpc* (KO) strains. The mRNA levels of *GAPDH2* was used as internal reference to normalize the levels of *TgCS1*, *TgCS2* or *TgPrpC* in different samples. Then, the levels of *TgCS1*, *TgCS2* or *TgPrpC* in WT strains were set as 1 to compare their levels between WT vs KO strains. Mean ± SEM of three independent experiments, each with three replicates. Student's t-tests.
