# Supplementary material for: Two enzymes contribute to citrate production in the mitochondrion of Toxoplasma gondii
Source: J Biol Chem. 2024 Jul 11;300(8):107565. doi: 10.1016/j.jbc.2024.107565 (PMC11359734; doi:10.1016/j.jbc.2024.107565)
Supplement: Supplemental Table S1 [file mmc7.docx]

**Table S1. Primers used in this study**

| **Primers** | **Sequences** | **Used for** |
| --- | --- | --- |
| KO-CS1-gRNA-Fw | GTGACCCTTGCTCTGCTTCG GTTTTAGAGCTAGAAATAGC | Constructing pSAG1-CAS9-sgCS1 to delete CS1 |
| KO-CS1-gRNA-Rv | CGAAGCAGAGCAAGGGTCAC AACTTGACATCCCCATTTAC |  |
| KO-PrpC-gRNA-Fw | GTTAGCTTGCCATCTGTCGC GTTTTAGAGCTAGAAATAGC | Constructing pSAG1-CAS9-sgPrpC to delete PrpC |
| KO-PrpC-gRNA-Rv | GCGACAGATGGCAAGCTAAC AACTTGACATCCCCATTTAC |  |
| Local-CS1-gRNA-Fw | GCGAGCATACACAGCTGTTC GTTTTAGAGCTAGAAATAGC | Constructing pSAG1-CAS9-sgCS1-loc for localization |
| Local-CS1-gRNA-Rv | GAACAGCTGTGTATGCTCGC AACTTGACATCCCCATTTAC |  |
| U5-CS1-Fw | CGACTCACTATAGGGCGAATACTGACCATACGCCGTCTTTG | Amplifying the 5’ homologous arm of *CS1* to construct pCS1::DHFR |
| U5-CS1-Rv | GATGTCTTCTGCGCGGGTTG AGTTCGTTCCTTCACGGTCAG |  |
| U5-PrpC-Fw | CGACTCACTATAGGGCGAAT CAGTCGTCGACTTTGAAGCG | Amplifying the 5’ homologous arm of *PrpC* to construct pPrpC::DHFR |
| U5-PrpC-Rv | GATGTCTTCTGCGCGGGTTGCTAAACCTGACAACGCTGAAGAG |  |
| U3-CS1-Fw | GCCACAAGTTCAGCGTGTCCATCAACCGAAGGTGTTCGAG | Amplifying the 3’ homologous arm of *CS1* to construct pCS1::DHFR |
| U3-CS1-Rv | GCTATGACCATGATTACGCC ACAACAGCTCGTTCCCTATC |  |
| U3-PrpC-Fw | GCCACAAGTTCAGCGTGTCC ACTTACCATTACACGAATCCG | Amplifying the 3’ homologous arm of *PrpC* to construct pPrpC::DHFR |
| U3-PrpC-Rv | GCTATGACCATGATTACGCC AATCCTAGACCCAGCGGCGTTC |  |
| DHFR-Fw | CAGGCTGTAAATCCCGTGAG | Amplifying DHFR to construct pCS1/ PrpC::DHFR |
| DHFR-Rv | GATTCCGTCAGCGGTCTGTC |  |
| pUC19-vector-Fw | GGCGTAATCATGGTCATAGC | Amplifying the pUC19 vector fragment to construct pCS1/ PrpC::DHFR |
| pUC19-vector-Rv | CTCGAATTCACTGGCCGTCG |  |
| Comp-CAT-HA-Fw | TATCCTTACGATGTTCCAGATT | Amplifying the Tub-CAT-HA vector from pComp-LDH1 |
| Comp-CAT-HA-Rv | CTTGCTCACCATTTTAGATC |  |
| CS1-PCR1-Fw | GCTCTGCACTTCCCACTTCC | PCR1 of *Δcs1::DHFR* |
| CS1-PCR1-Rv | CCTAGGGTCAAGTGGATCTTGG |  |
| CS1-PCR2-Fw | TCGGTAGCGACGAGGATATG | PCR2 of *Δcs1::DHFR* |
| CS1-PCR2-Rv | CTTACCTCCCTCGACATGTG |  |
| CS1-PCR3-Fw | GTGCATCCATTCTCAGTCGC | PCR3 of *Δcs1::DHFR* |
| CS1-PCR3-Rv | TGCAAGACCGTAGCTAGAATACC |  |
| PrpC-PCR1-Fw | TAGGTACCCGCATTACATCAAG | PCR1 of *Δprpc::DHFR* |
| PrpC-PCR1-Rv | CCTAGGGTCAAGTGGATCTTGG |  |
| PrpC-PCR2-Fw | TCGGTAGCGACGAGGATATG | PCR2 of *Δprpc::DHFR* |
| PrpC-PCR2-Rv | TAACCATGAGCCGTTGAATC |  |
| PrpC-PCR3-Fw | GACCCTGTGCGAACCCAGAG | PCR3 of *Δprpc:DHFR* |
| PrpC-PCR3-Rv | ATGGACGTAGCGTCGTTTGTG |  |
| Comp-CAT-TY-Fw | TATCCTTACGATGTTCCAGATT | Amplifying the tubulin promoter, CAT cassette and smHA from pCom-LDH1 |
| Comp-CAT-TY-Rv | CTTGCTCACCATTTTAGATC |  |
| GAPDH1-qPCR-Fw | TGAAGGGAATCATCAGCTACAC | qPCR for parasite load determination |
| GAPDH1-qPCR-Rv | TGTCAAACACGGAGGAGAAC |  |
| CS1-CDS-Fw | GGCTCACACAGAGAACAGATTGGT TGTGGCGGGCTCTCCAACG | Amplifying CS1 from cDNA of RH strain to construct pESUMO-CS1 |
| CS1-CDS-Rv | CAGTGGTGGTGGTGGTGGTG GTTGCCTTTCCTCTCAACGCATGC |  |
| PrpC-CDS-Fw | GGCTCACACAGAGAACAGATTGGTCAGTCTGGTGTGCACGTGCCTACAG | Amplifying PrpC from cDNA of RH strain to construct pESUMO-PrpC |
| PrpC-CDS-Rv | CAGTGGTGGTGGTGGTGGTG GAGCCGACTTCTGGACTGCTCAG |  |
| Comp-CS2 -Fw | GATCTAAAATGGTGAGCAAGATGAATCGCCTCAGTGTGATCAATG | Amplifying CS2 from gDNA of RH strain to construct pTub-CS2-HA-*DHFR* |
| Comp-CS2-Rv | TCTGGAACATCGTAAGGATAGATTTCGTTTGCCGTTAAGGCATTC |  |
