# Supplementary material for: Two enzymes contribute to citrate production in the mitochondrion of Toxoplasma gondii
Source: J Biol Chem. 2024 Jul 11;300(8):107565. doi: 10.1016/j.jbc.2024.107565 (PMC11359734; doi:10.1016/j.jbc.2024.107565)
Supplement: Supplemental Figure S1 [file mmc1.pdf]

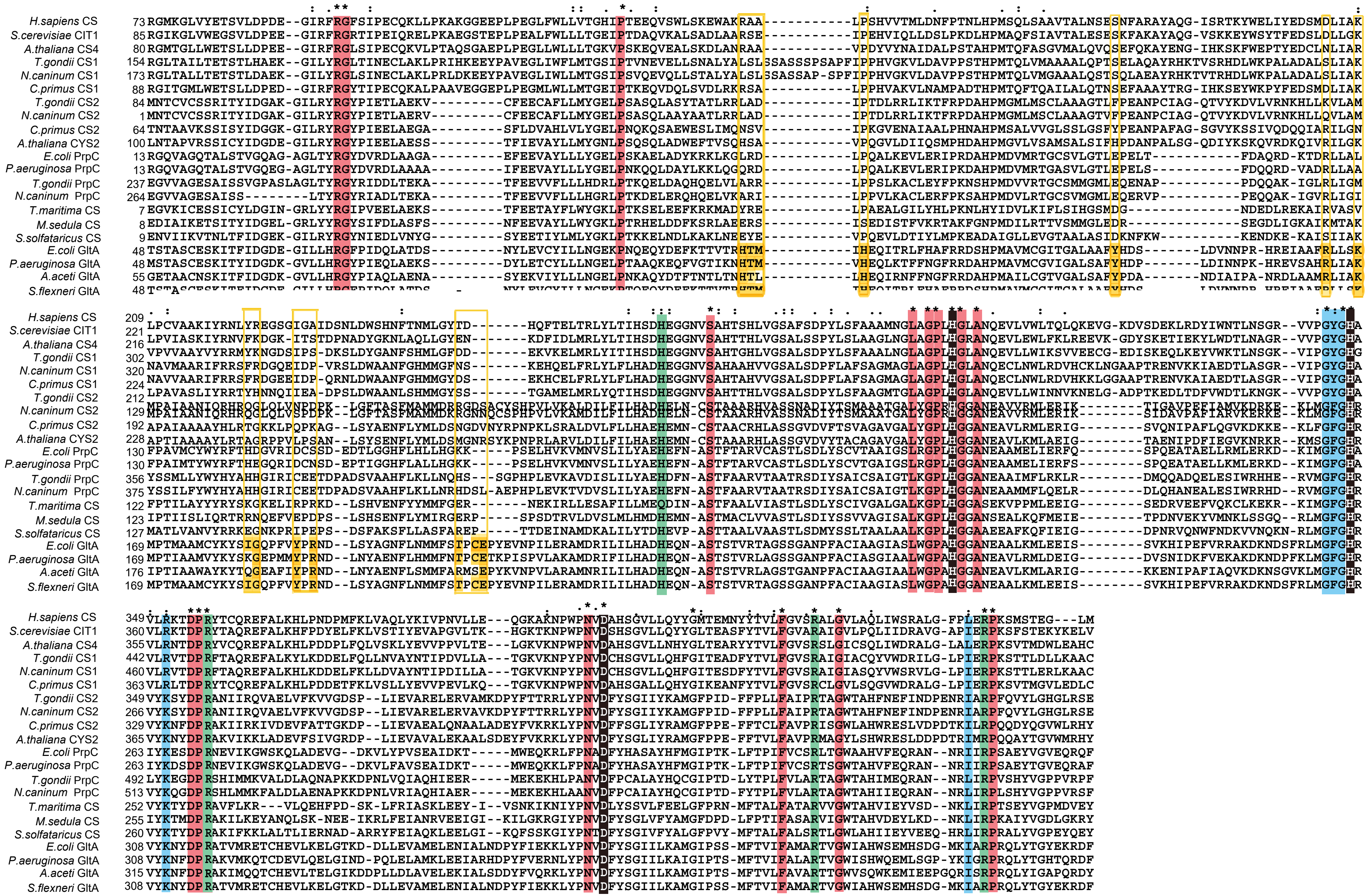

Figure S1. Sequence alignments of CS and related enzymes. Sequence alignment of the three putative citrate synthases from *Toxoplasma gondii* with known citrate synthases from representative species. Residues highlighted with red frames indicate conserved residues. Catalytic residues essential for CS activity are highlighted in black. Key residues for acetyl-CoA/CoA binding are marked in blue, while those involved in OAA/citrate binding are marked in green.
